# Orf virus DNA prime-protein boost strategy is superior to adenovirus-based vaccination in mice and sheep

**DOI:** 10.3389/fimmu.2023.1077938

**Published:** 2023-03-21

**Authors:** Yan Wang, Shihui Sun, Kui Zhao, Le Du, Xinyue Wang, Wenqi He, Feng Gao, Deguang Song, Jiyu Guan

**Affiliations:** Key Laboratory of Zoonosis, Ministry of Education, College of Veterinary Medicine, Jilin University, Changchun, China

**Keywords:** Orf virus, DNA prime-protein boost vaccination, DNA prime-adenovirus boost vaccination, immune responses, Orf immunization strategy

## Abstract

Contagious ecthyma (Orf), an acute and highly contagious zoonosis, is prevalent worldwide. Orf is caused by Orf virus (ORFV), which mainly infects sheep/goats and humans. Therefore, effective and safe vaccination strategies for Orf prevention are needed. Although immunization with single-type Orf vaccines has been tested, heterologous prime-boost strategies still need to be studied. In the present study, ORFV B2L and F1L were selected as immunogens, based on which DNA, subunit and adenovirus vaccine candidates were generated. Of note, heterologous immunization strategies using DNA prime-protein boost and DNA prime-adenovirus boost in mice were performed, with single-type vaccines as controls. We have found that the DNA prime-protein boost strategy induces stronger humoral and cellular immune responses than DNA prime-adenovirus boost strategy in mice, which was confirmed by the changes in specific antibodies, lymphocyte proliferation and cytokine expression. Importantly, this observation was also confirmed when these heterologous immunization strategies were performed in sheep. In summary, by comparing the two immune strategies, we found that DNA prime-protein boost strategy can induce a better immune response, which provides a new attempt for exploring Orf immunization strategy.

## 1 Introduction

Contagious ecthyma (Orf) is an acute and highly contagious zoonosis caused by Orf virus (ORFV). ORFV mainly affects goats, sheep and other animals like camels, dogs, cats, and squirrels. Humans are also susceptible to ORFV (1). At present, Orf is prevalent in almost all countries and regions where sheep are raised (2, 3). Orf disease is mainly characterized by the formation of papules, pustules and ulcers on the mucous membranes of the mouth, lips, tongue and nose of the affected animals (4). Although the mortality rates of adult sheep are low, lambs are susceptible and have a high incidence rate, reaching over 90%. Once Orf breaks out in sheep, it will cause substantial economic losses (5, 6). Therefore, developing a prophylactic vaccine against Orf is essential to control and prevent the transmission of this disease. ORFV is drought and desiccation tolerant and can survive in the environment for months to years. It can be transmitted to humans by direct or indirect contact. Orf is an occupational hazard that can infect people with long-term contact with sick animals, such as farmers, animal careers and veterinarians (7).

At present, vaccination is still considered as the main strategy to prevent and control Orf disease. There is an autologous vaccine for Orf, which is prepared by taking the scab from ORFV-infected animals and proved to be useful for controlling the outbreak of Orf, however, the scab from vaccinated lesions may cause potential environmental pollution (8). A German company used an Orf virus strain (Orf-V D1701), and passaged to attenuate it for preparing a live attenuated vaccine, which can protect ewes and lambs for 4-6 months (8). In UK, there are two kinds Orf vaccines for sale, Scabivax Orf vaccine produced by Mallinckrodt Veterinary Ltd and Vaxall Orf vaccine produced by Willows Francis Veterinary (9). However, none of these Orf vaccines have been reported to induce durable immunity in the animals (2, 9). Therefore, Viral vector vaccine, DNA and subunit vaccines are considered as promising candidate vaccines (8). In which, DNA (10) and submit (11) vaccination have been shown to induce both humoral and cellular immune. In the study of multiple viral vaccines, it is found that both types of vaccines can cause strong antibody response against pathogens (12–14).

ORFV is a double-stranded DNA virus, which belongs to the Poxviridae family, parapoxvirus genus. The genome of ORFV contains around 134 genes, and the central core region (ORFs 009-111) is highly conserved and plays critical roles in maintaining viral replication, maturation and morphogenesis (15). B2L and F1L genes are highly conserved, PCR is often used to detect the existence of these two genes in clinic to prove the infection of ORFV (16, 17). The ORFV B2L gene (ORF 011) and ORFV F1L gene (ORF 059) are two critical immunogenic genes that express 42 kDa and 37 kDa proteins, respectively. It is reported that these two immunogenic proteins could induce strong immune responses after vaccination and are considered as candidate antigens for developing vaccines (18, 19). For instance, F1L protein and its truncated form displayed good immunogenicity and induced antibody production (19, 20).

Interestingly, a research has shown that recombinant DNA vaccines containing ORF B2L-F1L chimeric gene could induce higher antibody levels in mice compared with single-gene DNA vaccines (21). However, the immune response triggered by single-type vaccine immunization still needs improvement. A recent study has reported that a heterologous Orf DNA vaccine priming-subunit vaccine boosting strategy induced higher immune responses than single-type vaccine immunization (22). Similarly, the efficacy of heterologous priming-boosting strategy has also been tested in preventing several diseases such as Newcastle disease virus, foot-and-mouth disease virus and middle east respiratory syndrome coronavirus (MERS-CoV) (13, 14, 23).

In the present study, we prepared DNA, adenovirus and subunit vaccines, containing B2L and F1L proteins as immunogens. Then DNA prime-protein boost, DNA prime-adenovirus boost and other single-type vaccine immunization strategy were evaluated in mice and sheep.

## 2 Materials and methods

### 2.1 Plasmids and cells

pDONR221-223-CBH-gcGFP, pAdenoG, and pcDNA3.1 (+) were all preserved in our laboratory. HEK293A and Primary ovine fetal turbinate (OFTu) cells were cultured in DMEM (Gibco, Grand Island, USA) supplemented with 10% fetal bovine serum (FBS), penicillin (100 U/mL) and streptomycin (100 mg/mL) at 37°C and 5% CO_2_.

### 2.2 Construction of recombinant adenoviruses plasmids

Based on the ORFV B2L-P2A-F1L PCR product from our previous studies (22), forward and reverse primers for B2L and F1L gene sequences were designed and added appropriate restriction sites as follows: 011-F: 5′- CTTTGTACAAAAAAGTTGGCCCTAGGGCCACC ATGTGGCCGTTCTCCTCCATC; 059-R: CATTTGTCGTCATCATCCTTATAGTCGAC TTATCACACGATGGCCGTGACC. Then, the amplified fusion gene was cloned into pDONR221-223-CBH-gcGFP shuttle plasmid using Tranzyme MasterMix (abm, Zhenjiang, China), and the fusion gene was subsequently transferred into pAdenoG vector using Fuzyme Cloning Kit (abm, Zhenjiang, China) to construct a recombinant adenoviruses vector named rAdV-B2L-P2A-F1L. The pAdenoG as the control adenovirus vector.

### 2.3 Construction, packaging, and purification of recombinant adenovirus

To produce the recombinant virus. The HEK293A cells were passaged into T25 cell culture flasks and incubated at 37°C in 5% CO_2_. When the cell confluence is about 70-80%, the recombinant adenovirus plasmid was digested with restriction endonuclease PacI (NEB, Boston, USA), and 4 μg recombinant plasmid was transfected into HEK293A cells using Lipofectamine 3000 (Invitrogen, Carlsbad, USA) transfection reagent, and the cytopathic effect (CPE) was observed. When the cells showed CPE, the supernatant was collected, which was the F1 generation adenovirus, and the F1 generation virus was taken to infect 293A cells. When about 80% of the cells were diseased, the virus was collected and continued to be transmitted to the F3 generation. Until the 20th generation of recombinant adenovirus, the virus DNA was extracted with the innuPREP Virus DNA Kit (Analytik, Jena, Germany) as a template for identification. Adenovirus was purified from HEK293A cell extract by CsCl (Sigma, Maryland, Germany) density gradient centrifugation. CsCl was removed by size exclusion chromatography using a PD-10 column (GE Healthcare, Pittsburgh, UK) and the adenovirus was stored in an adenovirus storage buffer (10 mM Tris, pH 8.0, 2 mM MgCl_2_, 5% sucrose) at -80°C. IFU/mL = [(number of positive signals/each field of vision) × (Number of visual fields/each cell pore)] ÷ [Amount of virus added (mL) × (dilution ratio)].

### 2.4 Western blotting of recombinant adenovirus

OFTU cells were infected with recombinant adenovirus (MOI = 100) cells were harvested after 48 h, PMSF and RIPA lysis buffer (Beyotime, Shanghai, China) were mixed (1:100) and added separately to prevent protein degradation. Proteins were electrophoresed on a 10% separating gel; proteins were transferred to nitrocellulose membranes; mouse polyclonal antibodies (22) in the ratio of 1:500 or GAPDH mouse monoclonal antibody (1:5000) (Proteintech, Chicago, USA) was used as the primary antibody and incubate overnight at 4°C. HRP-conjugated goat anti-mouse IgG (1:5000) (Abcam, Cambridgeshire, UK) was used as the secondary antibody and incubated at 37°C for 1 h. Record results using a gel imaging system (Tanon, Shanghai, China).

### 2.5 Preparation of DNA and subunit vaccine

The fusion gene also was cloned into pcDNA3.1 (+) to construct a DNA vaccine vector named 3.1-B2L-P2A-F1L. In our previous study, it has been demonstrated *in vitro* that this fusion gene can be expressed in eukaryotic cells OFTu. The Purified B2L and F1L proteins as subunit vaccine were described in our previous article (22), the identified recombinant plasmid pET-32a-B2L and pET-30a-cF1L were transformed into BL21(DE3) pLysS chemically competent cell; monoclonal colonies in solid medium were picked up and added to 4 mL liquid LB medium for culture; after 12 h, the total starter culture was transferred into 400 mL fresh liquid LB medium; when OD_600_ value was reached 0.4-0.6, isopropyl β-D-thiogalactoside (IPTG) with a final concentration of 0.2 mM was added to induce for 12 h; the bacterial solution was centrifuged at 8000 rpm for 10 min; then, the soluble binding buffer (20 mM pH7.9 Tris-HCl, 10 mM imidazole, 0.5M NaCl) was used to resuspended mycelium, lysed by sonication under the condition of ice water mixture; the suspension was centrifuged at 8000 rpm for 10 min at 4°C; the supernatant and sediment were collected for detection of soluble and inclusion body proteins; B2L protein was identified as inclusion body protein, and truncated cF1L protein was soluble protein; the inclusion body binding buffer (20 mM pH7.9 Tris-HCl, 5 mM imidazole, 0.5 M NaCl, 8 M urea) was used to dissolved the inclusion body protein; load all supernatant or dissolved the inclusion body protein into Ni column (ComWin, Beijing, China); finally, the B2L bound protein was eluted with elution buffer (20 mM pH7.9 Tris-HCl, 500 mM imidazole, 0.5M NaCl, 8 M urea), the F1L bound protein was eluted with elution buffer (20 mM pH7.9 Tris-HCl, 500 mM imidazole, 0.5 M NaCl) and analyzed by 10% SDS-PAGE or western blotting. The concentration of purified B2L and F1L proteins was 1 mg/mL and 2 mg/mL, respectively, which was detected by BCA (Beyotime, Shanghai, China).

All vaccines were mixed in a volume of 1:1 with complete Freund’s adjuvant for the first immunization; in all vaccine immunization groups, booster immunized were mixed in a volume of 1:1 with incomplete Freund’s adjuvant except rAdV vaccine and DNA/rAdV vaccine groups, shaken and emulsified at 4°C overnight to prepare a water-in-oil emulsion.

### 2.6 Animals

6-8 weeks old female BALB/c mice (n = 70) were purchased from the Center of Experimental Animals Changsheng Biotechnology Co., Ltd. in Liaoning, China. Sheep (n = 15) were purchased from Longsheng Experimental Animal Science and Technology Co., Ltd. in Jilin Changchun, China. All animals in the experiment were raised under specific-pathogen-free conditions in a light/dark cycle of about 12 h; temperature was around 27°C; humidity was around 42%, with food and water ad libitum. All animal experiments were conducted according to the Guidelines for the Care and Use of Research Animals provided by Jilin University. Institutional Review Board Statement: All animal experiments were approved by the Institutional Animal Care and Use Committee of the College of Veterinary Medicine, Jilin University, China.

### 2.7 Animal immunization

Seven groups of BALB/c mice (n = 10/per group) were immunized twice at 21-day intervals. Mice were vaccinated with the DNA vaccines (3.1-B2L-P2A-F1L plasmids; 100 μg each); subunit vaccine (B2L/F1L proteins, the total of 40 μg each); recombinant adenovirus (rAdV) vaccine (rAdV-B2L-P2A-F1L virus, 1 × 10^9^ PFU each); the combination of DNA vaccine (3.1-B2L-P2A-F1L plasmids; 100 μg each) and rAdV vaccine (rAdV-B2L-P2A- F1L virus, 1 × 10^9^ PFU each); the combination of DNA vaccine (3.1-B2L-P2A-F1L plasmids; 100 μg each) and subunit vaccine (B2L/F1L proteins, the total of 40 μg each); The control group mice injected with PBS (100 μL each) and pAdenoG (an empty adenovirus; 1 × 10^9^ PFU each) separately. In the experiment, the immune mode of DNA and adenovirus vaccine is intramuscular injection, and the immune mode of the subunit vaccine is a subcutaneous injection. The details as shown in **Table 1**.

**Table 1:** Immunization strategies.

Animals	Groups	Prime (0 day)	Boost (21 days)	Immune dose
Mice (n = 10)	PBS	PBS	PBS	100/100 μL
	pAdenoG	pAdenoG adenovirus	pAdenoG adenovirus	1×10^9^/1×10^9^ PFU
	I-ORFV	Inactivated ORFV	Inactivated ORFV	1×10^5^/1×10^5^TCID_50_
	DNA/DNA	3.1-B2L-P2A-FIL	3.1-B2L-P2A-FIL	100/100 μg
	Protein/Protein	B2L and cF1L proteins	B2L and cF1L proteins	20+20/20+20 μg
	rAdV/rAdV	rAdV-B2L-P2A-FIL	rAdV-B2L-P2A-FIL	1×10^9^/1×10^9^ PFU
	DNA/rAdV	3.1-B2L-P2A-FIL	rAdV-B2L-P2A-FIL	100 μg/1×10^9^ PFU
	DNA/Protein	3.1-B2L-P2A-FIL	B2L and cF1L proteins	100 μg/20+20 μg
Sheep (n = 5)	PBS	PBS	PBS	100/100 μL
	DNA/rAdV	3.1-B2L-P2A-FIL	1×10^9^ PFU	500 μg/1×10^11^ PFU
	DNA/Protein	3.1-B2L-P2A-FIL	B2L and cF1L proteins	500 μg/100+100 μg

Three groups of sheep (n = 5/per group) were immunized twice *via* intramuscular injection at 21-day intervals. Sheep were injected with PBS (1 mL each) as the control group; the combination of DNA vaccine (3.1-B2L-P2A-F1L plasmids; 500 μg each) and rAdV vaccine (rAdV-B2L-P2A-F1L virus, 1 × 10^11^ PFU each); the combination of DNA vaccine (3.1-B2L-P2A-F1L plasmids; 500 μg each) and subunit vaccine (B2L/F1L proteins, the total of 200 μg each). The details as shown in **Table 1**.

### 2.8 Specific antibody detection

Serum samples from immunized mice and sheep were collected on days 0, 7, 14, 21, 28, and 35 after primary immunization. Serum IgG antibodies specific for ORFV B2L and F1L were detected using an indirect enzyme-linked immunosorbent assay (ELISA). The purified 3 µg/mL ORFV 011 and 059 proteins (100 µL per well) were diluted with 0.05 M carbonate buffer (pH 9.6) and coated overnight at 4°C; the next day, the plates were washed three times with phosphate-buffered saline (PBS) containing 0.1% Tween-20 (PBS-T), blocked with 5% non-fat milk in PBS-T for 1 h at 37°C; the plates were washed with PBS-T, the mouse serum was diluted to 1:100 and then 2-fold multiple dilutions. 100 μL of serum diluted was added to each well and incubated at 37°C for 1 h; the plates were washed with PBS-T, the secondary antibodies of goat anti-mouse IgG or rabbit anti-sheep IgG antibody conjugated with HRP (Proteintech, Chicago, USA) which diluted 1:5000 was added and incubated at 37°C for 1 h; the plates were washed with PBS-T, added 100 μL of two-component TMB chromogenic solution (Solarbio, Beijing, China) and incubated for 15 min in the dark at RT; Finally, 50 μL of 2 M H_2_SO_4_ was added to stop the reaction; the optical density (OD) was measured at 450 nm by a microplate reader. Values 2.1-fold higher than the control group were considered to be positive. The end-point titer was determined as the highest dilution.

### 2.9 Specific antibody IgG subclass detection

IgG1 and IgG2a expression in mouse serum were detected by goat anti mouse IgG1, IgG2a secondary antibody (Sigma, Saint Louis, USA); IgG1 and IgG2a expression in sheep serum were detected by sheep IgG1 and IgG2a ELISA Kit (Jiang Lai, Shanghai, China), and all of them detected according to the reagent instructions.

### 2.10 Spleen lymphocyte proliferation assay

On the 14th day after the boost immunization, the mice spleen was aseptically removed, ground using a 10 mL syringe, and filtered through a cell mesh filter to prepare a single-cell suspension. The spleen lymphocytes were isolated by mouse lymphocyte separation medium (Solarbio, Beijing, China), added an appropriate amount of separating liquid was put into the centrifuge tube, and spread the cell suspension over the liquid level of the separating liquid; 1000 g, centrifugation for 30 min; after centrifugation, there has been an obvious stratification, the white membrane layer between the plasma and the separating liquid is the lymphocyte layer, carefully sucked lymphocyte cells into 15 mL clean centrifuge tube, and washed lymphocyte cells with 10 mL PBS. 250 g, centrifugation for 10 min; discarded the supernatant, resuspension cells with 5 mL PBS, 250 g, centrifugation for 10min. The cell concentration was adjusted to 1 × 10^6^/ml with 10% RPMI 1640 (10% FBS, 2 mM L-glutamine, 100 U/ml of penicillin, 100 μg/ml of streptomycin) complete medium (Gibco, Grand Island, USA), and the cells were mixed and added to a 96-well plate, add 100 μL of cell suspension to each well. 5 μg/mL purified B2L and cF1L proteins were used as specific antigens, 1640 complete medium was used as a negative control, and ConA was stimulated at a final concentration of 5 μg/mL as a positive control. After 68 h, 10 μL MTT (5 mg/mL) was added to each well and cultured for 4 h, removed the supernatant, added 100 μL DMSO into each well, and placed in the dark at RT for 15 min. The OD value of each well was determined at 450 nm.

### 2.11 Flow cytometry

To detect changes in specific T cell subsets in sheep after vaccine immunization. On days 0 and 35, blood collection from sheep jugular vein, and the erythrocytes were lysed with erythrocyte lysate (Solarbio, Beijing, China); the cells were washed three times with PBS; FITC-conjugated anti-sheep CD4 (AbD Seratec, Kidlington, Britain), PE-conjugated anti-sheep CD8a (AbD Seratec, Kidlington, Britain) were incubated at 4°C for 30 min; washed cells 3 times with PBS; finally resuspended in flow buffer. Antibody-stained cells were analyzed on a BD FACSAria II flow cytometer using FlowJo software (Tree Star, Ashland, OR, USA).

### 2.12 Cytokine detection

On day 14, after the boost immunization, the production of cytokines was determined using splenocytes from immunized groups. The spleen cells were stimulated with 5 μg/mL purified B2L and cF1L proteins and the cultures were collected after 48 h and kept at -80°C pending assaying of the cytokines. Cytokines in the culture supernatant were quantified using mouse IL-2, IL-4, IL-6, IFN-γ, and TNF-α ELISA Kits (Biolegend, San Diego, CA, USA) following the manufacturer’s instructions.

On day 14, after the boost immunization, the levels of cytokines production in sheep serum were determined using sheep IL-2, IL-4, IL-6, IFN-γ, and TNF-α ELISA Kits (Jiang Lai, Shanghai, China) following the manufacturer’s instructions.

### 2.13 Statistical analysis

All experimental data were performed at least three times. Data analysis was performed using t-test and one-way ANOVA in GraphPad Prism 6.0 software (San Diego, CA, USA); the data are presented as the means ± standard deviation (SD), and statistical significance was assumed when the P value was < 0.05.

## 3 Results

### 3.1 Production and identification of B2L and F1L expressing recombinant adenovirus

**Figure 1:**
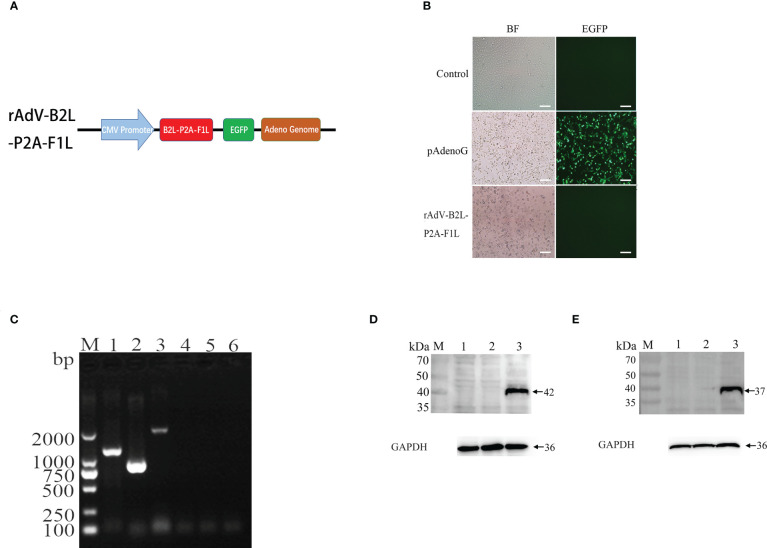
Production and identification of recombinant adenovirus. **(A)** Schematic diagram of rAdV-B2L-P2A-F1L plasmid. **(B)** Production of recombinant adenovirus after transfection of the recombinant plasmid into HEK293A cells. The left fields are shown in bright-field microscopy, and the right fields are shown in fluorescence microscopy. The magnification is 100 ×, Scale bars: 100 μm. **(C)** RT-PCR detected the presence of the B2L/F1L gene in the virus DN. Lane 1-3: the viral DNA of recombinant adenovirus rAdV-B2L-P2A-F1L was used as a template to amplify ORFV B2L (1137 bp), ORFV F1L (1011 bp), and ORFV B2L-P2A-F1L (2205 bp) genes, respectively. Lane 4-6: the viral DNA of pAdenoG adenovirus was used as a template and as a negative control to amplify ORFV B2L, ORFV F1L, and ORFV B2L-P2A-F1L genes, respectively. M: DL2000 DNA Marker. **(D)** Western blotting analysis expression of B2L gene in recombinant adenovirus infected ORFV cells. Membrane incubated with anti-B2L protein mouse polyclonal antibodies. Lane 1: OFTu cell lysates, lane 2 and lane 3: the infected pAdenoG adenovirus and rAdV-B2L-P2A-F1L recombinant adenovirus OFTu cell lysates. **(E)** Western blotting analysis expression of F1L gene in recombinant adenovirus infected ORFV cells. Membrane incubated with anti-cF1L protein mouse polyclonal antibodies. Lane 1: OFTu cell lysates, lane 2 and lane 3: the infected pAdenoG adenovirus and rAdV-B2L-P2A-F1L recombinant adenovirus OFTu cell lysates. M: protein molecular weight marker.

To generate recombinant adenovirus expressing B2L/F1L fusion protein, the recombinant plasmid of rAdV-B2L-P2A-F1L was constructed (**Figure 1A**). The PacI linearized recombinant plasmids pAdenoG and rAdV-B2L-P2A-F1L were transfected into HEK293A cells. The cytopathic effect (CPE) was observed about 14 days after transfection (**Figure 1B**). Compared with the control group, both HEK293A cells in pAdenoG adenovirus vector group and recombinant adenovirus rAdV-B2L-P2A-F1L group showed roundness and abscission. Under fluorescence microscope, HEK293A cells of adenovirus vector group showed green fluorescence. Purification of recombinant adenovirus by cesium chloride density gradient centrifugation, and the presence of the B2L/F1L gene in the virus DNA was detected by RT-PCR (**Figure 1C**). To detect whether B2L/F1L fusion protein could be independently expressed in OFTu cells under the cleavage of P2A peptide, OFTu cells were infected with recombinant adenovirus (MOI = 100) and were collected after 24 h. Western blotting was performed to examine the protein level in the cell lysates (**Figure 1D, E**). The B2L protein (42 kDa) could be detected when the anti-B2L mouse polyclonal antibodies were incubated with the lysates from recombinant adenovirus rAdV-B2L-P2A-F1L-infected cells (**Figure 1D**); The F1L protein (37 kDa) could be detected when anti-cF1L mouse polyclonal antibodies were incubated with the lysates from recombinant adenovirus rAdV-B2L-P2A-F1L infected OFTu cells (**Figure 1E**). No obvious protein band was found in non-infected or recombinant adenovirus pAdenoG infected OFTu cells. The results showed that the B2L and F1L proteins could be independently expressed in recombinant adenovirus rAdV-B2L-P2A-F1L-infected OFTu cells.

### 3.2 DNA prime-protein boost strategy induces higher specific antibody levels than DNA prime-rAdV boost strategy in mice

**Figure 2:**
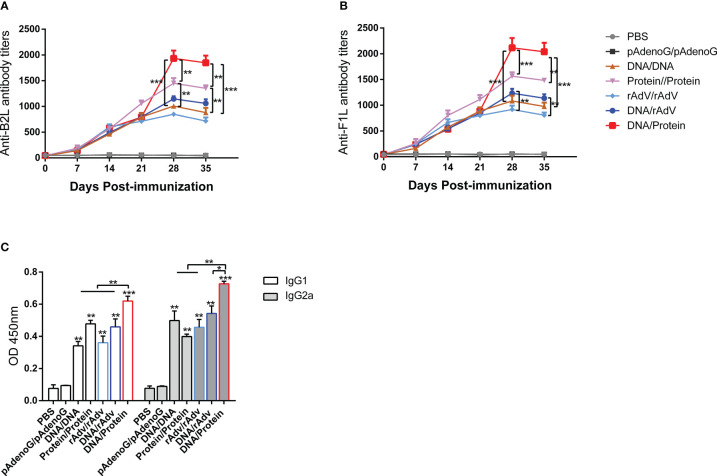
The levels of specific antibodies and IgG1 and IgG2a isotypes after vaccination in mice sera. **(A)** End-point titers of anti-B2L antibodies in mice were assayed by ELISA. **(B)** End-point titers of anti-F1L antibodies in mice were assayed by ELISA. **(C)** The levels of IgG1 and IgG2a antibody in mice sera. Sera were collected 14 days after the boost immunization for antibody detection, and they were measured by ELISA. Each group of data is expressed as mean ± standard error (n = 10). The OD value was determined at 450 nm. *p < 0.05, **p < 0.01, and ***p < 0.001.

We have previously constructed Orf DNA vaccine expressing B2L/F1L fusion protein and subunit vaccines with B2L and cF1L proteins (22). Then, we evaluated the capacity of different vaccines that could induce a humoral response in mice. The end-point titer of B2L and F1L-specific IgG in the mice serum was examined through indirect ELISA on days 0, 7, 14, 21, 28 and 35 after the first immunization (**Figure 2**). The results showed that all immunized mice displayed significant humoral responses (*p* < 0.0001) when compared with the PBS and pAdenoG groups from day 7 after the boost immunization; the B2L (**Figure 2A**) and F1L (**Figure 2B**) specific IgG titers in the DNA/Protein group were the highest, which was significantly higher (*p* < 0.01 or *p* < 0.001) than those from the DNA/DNA, Protein/Protein, rAdV/rAdV and DNA/rAdV groups, it can reach 1:1933 and 1:2116 respectively. Notably, on day 35, although the end-point titer of B2L and F1L-specific IgG in all vaccine-immunized mice was slightly lower than on day 28, the B2L and F1L-specific IgG titers in the DNA/Protein group were the highest, which was significantly higher (*p* < 0.01 or *p* < 0.001) than those of the DNA/DNA, Protein/Protein, rAdV/rAdV and DNA/rAdV groups, it can reach 1:1850 and 1:2026 respectively. As expected, control mice immunized with pAdenoG or PBS had no specific antibodies. Of note, the data indicate that DNA prime-protein boost immunization strategy could induce stronger specific antibodies than both rAdV/rAdV and DNA/rAdV immunization strategy.

On day 14, after the boost immunization, the levels of specific antibodies IgG subclasses IgG1 and IgG2a were examined by indirect ELISA, as shown in **Figure 2C**. The IgG1 and IgG2a levels of all vaccine immunization groups were significantly higher (*p* < 0.01 or *p* < 0.001) than the PBS and the pAdenoG groups; of note, the IgG1 and IgG2a levels from DNA/Protein group mice were significantly higher than those of the DNA/DNA (*p* < 0.01), Protein/Protein (*p* < 0.01), rAdV/rAdV (*p* < 0.01) and DNA/rAdV groups (*p* < 0.05 or *p* < 0.01); In addition, the IgG2a: IgG1 ratio of all vaccine-immunized groups were higher than 1 except Protein/Protein group. These results indicate that most vaccine immunization mainly induced Th1-type immune responses.

### 3.3 Evaluation of cellular immune responses in mice

**Figure 3:**
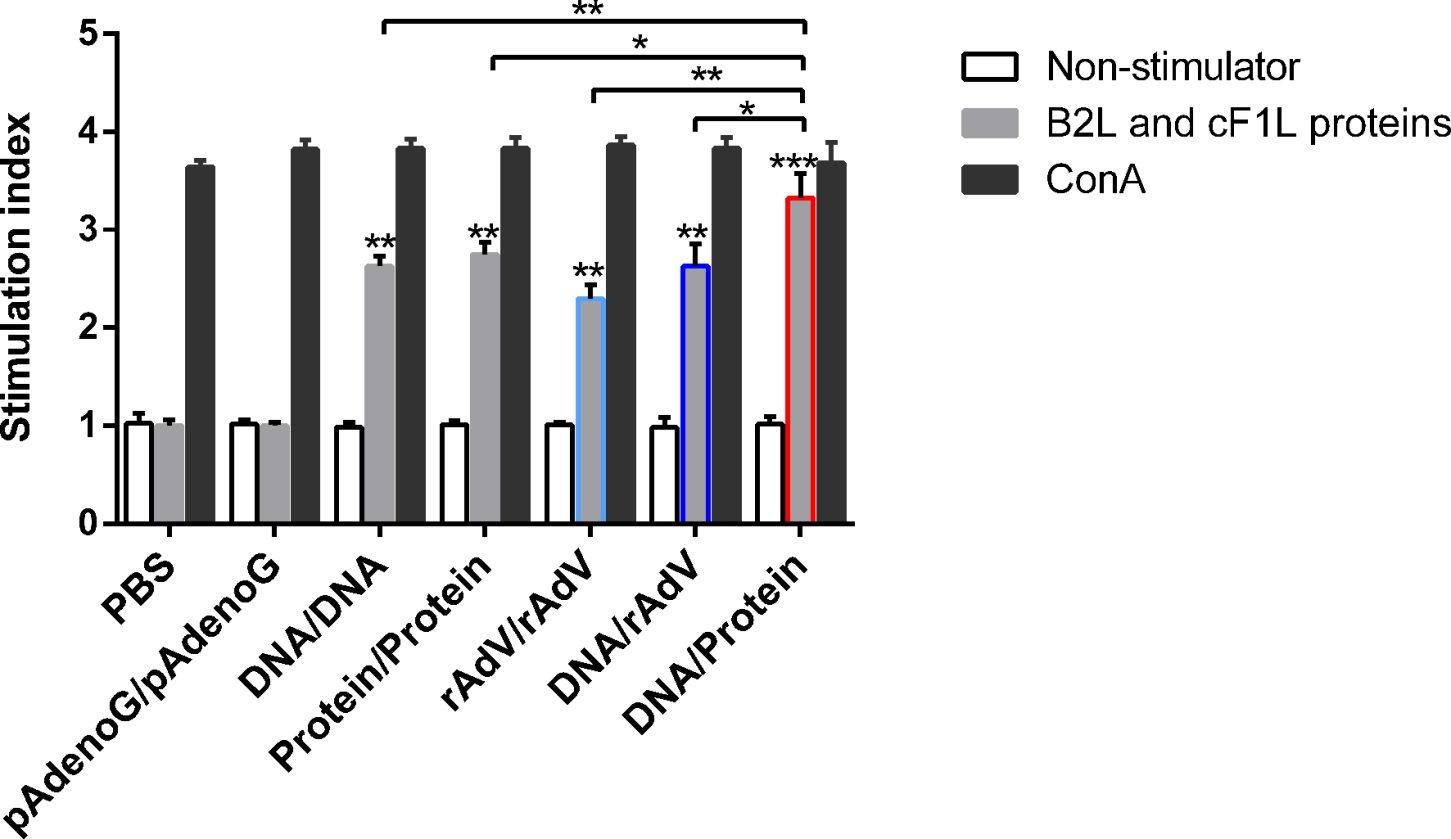
Lymphocyte proliferative responses in mice with different vaccine-immunized groups at 14 days after the boost immunization. Mice lymphocytes were isolated and stimulated with purified B2L and F1L (5 μg/mL) proteins. Lymphocyte proliferation response was measured and expressed as stimulation index (SI). SI = OD experimental group-OD background/OD negative control group-OD background. Each group of data is expressed as mean ± standard error (n = 5), *p < 0.05, **p < 0.01, and ***p < 0.001.

To evaluate the cellular immune response after the vaccination, the lymphocyte proliferative response was examined. The mice were euthanized on the 14th day after the boost vaccination, and splenocytes were isolated from the spleen of the mice and measured by MTT colorimetry. As shown in **Figure 3**, the stimulation index (SI) of mice splenocytes in all immunized groups were significantly higher (*p* < 0.01 or *p* < 0.001) than those of the PBS and pAdenoG groups; The SI value of DNA/Protein group was the highest among all immunization groups. Of note, it was significantly higher than those of the DNA/DNA (*p* < 0.01), rAdV/rAdV (*p* < 0.01), Protein/Protein (*p* < 0.05), and DNA/rAdV groups (*p* < 0.05). These results proved that the DNA prime-protein boost strategy induces higher proliferation of splenic lymphocytes compared to other vaccines, especially rAdV-based immunization.

### 3.4 Cytokine responses in vaccinated mice

**Figure 4:**
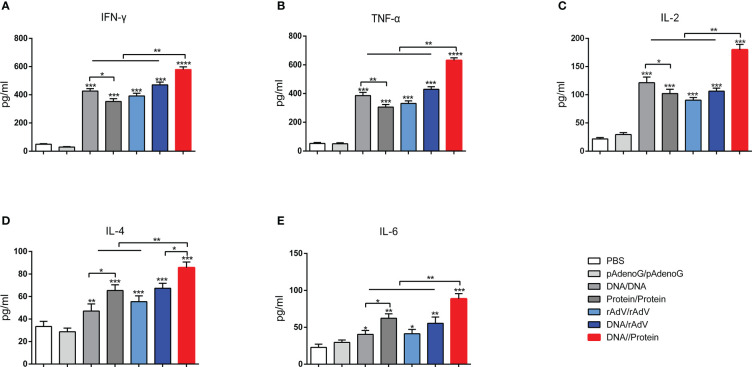
Production of various cytokines in culture supernatants of splenocytes. IFN-γ **(A)**, TNF-α **(B)**, IL-2 **(C)**, IL-4 **(D)**, and IL-6 **(E)** were measured using ELISA Kit. Data are expressed as mean ± standard error (n = 10). *p < 0.05, **p < 0.01, ***p < 0.001, and ****p < 0.0001.

On day 14, after the boost immunization, the cytokine concentrations in the supernatant of splenic lymphocytes were measured by ELISA, as shown in **Figure 4**. The levels of Th1-type cytokines (IFN-γ, TNF-α, and IL-2) (**Figures 4A–C**) in the group immunized with vaccines showed significantly higher levels (*p* < 0.001 or *p* < 0.0001) than the groups immunized with PBS and pAdenoG. The DNA/Protein group had highest IFN-γ, TNF-α, and IL-2 levels than (*p* < 0.01) those of the DNA/DNA, Protein/Protein, rAdV/rAdV, and DNA/rAdV groups. The IFN-γ, TNF-α, and IL-2 levels in the DNA/DNA group were significantly higher (*p* < 0.05 or *p* < 0.01) than in the Protein/Protein group.

Moreover, as shown in **Figures 4D, E**, Th2-type cytokine concentration of IL-4 and IL-6 among the vaccine immunization groups showed significantly higher (*p* < 0.05, *p* < 0.01, and *p* < 0.001) than those of the groups immunized with PBS and pAdenoG. The DNA/Protein group had significantly higher IL-4 levels than those of the DNA/DNA (*p* < 0.01), Protein/Protein (*p* < 0.01), rAdV/rAdV (*p* < 0.01), and DNA/rAdV groups (*p* < 0.05); Higher IL-6 levels in the DNA/Protein group were found compared to the DNA/DNA, Protein/Protein, rAdV/rAdV and DNA/rAdV groups (*p* < 0.01). IL-4 and IL-6 levels in the Protein/Protein were significantly higher than (*p* < 0.05) that of the DNA/DNA group. The above data demonstrate that DNA prime-protein boost strategy induces higher levels of cytokine responses compared to the immunization with rAdV-based immunization.

### 3.5 Levels of specific antibodies induced by two heterologous immunization strategies in sheep

**Figure 5:**
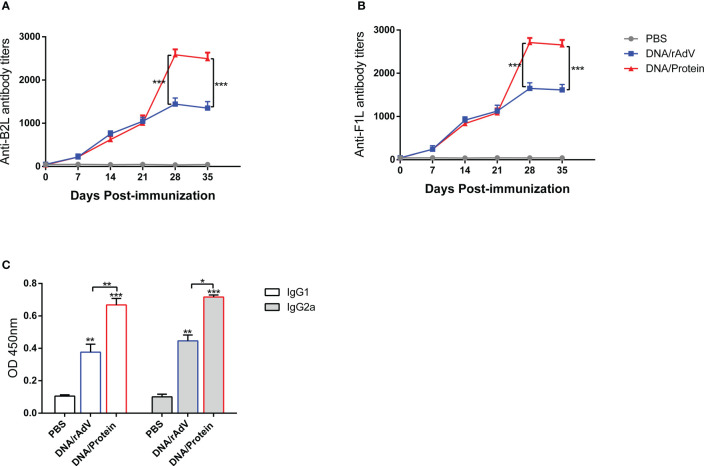
The levels of specific antibodies and IgG1 and IgG2a isotypes after vaccination in sheep sera. **(A)** End-point titers of anti-B2L antibodies in sheep were assayed by ELISA. **(B)** End-point titers of anti-F1L antibodies in sheep were assayed by ELISA. **(C)** The levels of IgG1 and IgG2a antibody in sheep sera. Sera were collected 14 days after the boost immunization for antibody detection, they were measured by ELISA. Each group of data is expressed as mean ± standard error (n = 5). The OD value was determined at 450 nm. *p < 0.05, **p < 0.01, and ***p < 0.001.

To evaluate the humoral response elicited by the two heterologous immunization strategies in sheep, we chose to perform the immunization with both DNA prime-protein vaccine boost and DNA prime-rAdV boost strategies. Serum samples were collected at days 0, 7, 14, 21, 28 and 35 after the first immunization, and the end-point titer of B2L and F1L-specific IgG in the sheep serum was measured by indirect ELISA method as shown in **Figure 5**. From day 7 after the booster immunization, the B2L and F1L-specific IgG titers in the DNA/Protein and DNA/rAdV groups were significantly higher (*P* < 0.0001) when compared with the PBS group. Importantly, the B2L and F1L-specific IgG titers from DNA/Protein immunized sheep were significantly higher (*P* < 0.001) than that from DNA/rAdV group, which was in line with the observation in mice, it can reach 1: 2583 and 1: 2710 respectively. In addition, on day 35, the B2L and F1L-specific IgG titers in the DNA/Protein group was also the highest, which was significantly higher (*p* < 0.001) than that of the DNA/rAdV group, it can reach 1:2493 and 1:2656 respectively. No corresponding specific antibodies were detected in the PBS group throughout the experiment. The above data suggested that both kinds of heterologous immunization strategies induce a high humoral response in sheep, and the DNA prime-protein boost vaccination strategy shows a stronger humoral immune response.

On day 14, after the boost immunization, the levels of specific antibodies IgG subclasses IgG1 and IgG2a were examined by ELISA Kit, as shown in **Figure 5**. The IgG1 and IgG2a levels in DNA/rAdV and DNA/Protein groups were significantly higher (*p* < 0.01 or *p* < 0.001) than that of the PBS group. Of note, the IgG1 and IgG2a levels in the DNA/Protein immunized sheep were significantly higher than that of the DNA/rAdV group (*p* < 0.05 or *p* < 0.01); Moreover, the ratio of IgG2a: IgG1 in the two vaccine-immunized groups were higher than 1. These results indicated that DNA prime-protein boost-induced Th1-type cytokine immune response is higher than the induction by DNA prime-rAdV boost strategies.

### 3.6 Subsets of specific T lymphocyte cells

**Figure 6:**
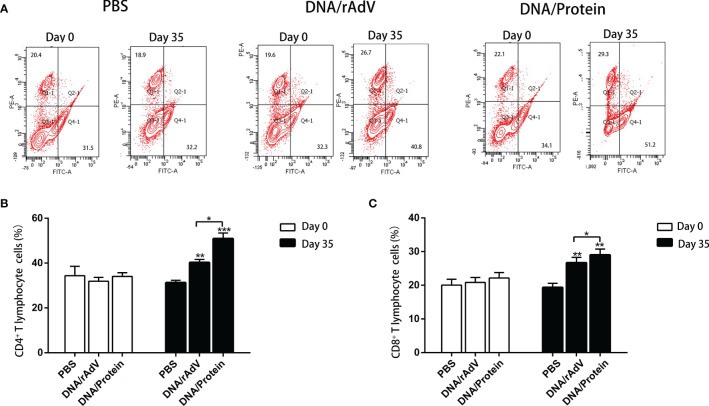
The detection of CD8^+^ and CD4^+^ T cells in the peripheral blood of sheep after different prime-boost immunization strategies by flow cytometry assay. **(A)** Representative four-quadrant dot blots for the CD4^+^ and CD8^+^ T cell ratio in PBS, DNA/rAdV and DNA/Protein groups before immunization on day 0 and day 35. **(B)** Data statistics and analysis of CD4^+^T cells. **(C)** Data statistics and analysis of CD8^+^ cells. Data are expressed as mean ± standard error (n = 5). *p < 0.05, **p < 0.01 and ***p < 0.001.

On day14, after boost immunization, to examine whether or not immunized sheep by two heterologous immunization strategies could induce a T-cell response. We analyzed the percentage of CD4^+^ and CD8^+^ T cells in the peripheral blood of sheep by flow, as shown in **Figure 6**. On day 35, the proportion of CD4^+^ and CD8^+^ T cells in the DNA/Protein and DNA/rAdV groups were significantly higher than that in the PBS control group (*P* < 0.01 or *P* < 0.001); the proportion of CD4^+^ and CD8^+^ T cells in the DNA/Protein group was significantly higher than (*P* < 0.05) that in the DNA/rAdV group. These results indicate that the DNA Prime-Protein boost strategy could induce a T-cell response in sheep.

### 3.7 Cytokine responses in vaccinated sheep

**Figure 7:**
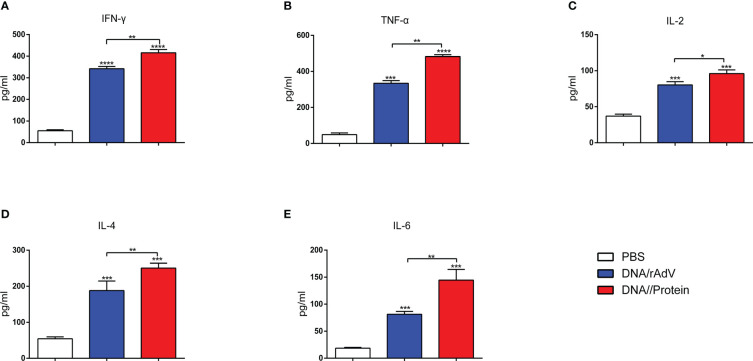
Sera were analysed for the levels of IFN-γ **(A)**, TNF-α **(B)**, IL-2 **(C)**, IL-4 **(D)**, and IL-6 **(E)** at 14 days after the boost immunization. The serum samples were tested using ELISA kits. Data are expressed as mean ± standard error (n = 5). *p < 0.05, **p < 0.01, ***p < 0.001, and ****p < 0.0001.

On day14, after the boost immunization, to further identify the Th1-type cytokines (IFN-γ, TNF-α, and IL-2) and Th2-type cytokines (IL-4 and IL-6) produced in response to the vaccines, as shown in **Figure 7**. The data revealed that IFN-γ, TNF-α, and IL-2 levels immunized with vaccines were significantly higher than (*p* < 0.001, *p* < 0.0001) that immunized with PBS. Notably, Th1-type cytokines from DNA/Protein immunized sheep induced the highest levels compared to the DNA/rAdV (*p* < 0.05, *p* < 0.001) immunized sheep (**Figures 7A–C**).

In addition, the IL-4 and IL-6 levels in the DNA/Protein and DNA/rAdV groups were significantly higher (*p* < 0.001) than the PBS group. Importantly, IL-4 and IL-6 levels from DNA/Protein immunized sheep elicited significantly higher than (*p* < 0.01) those from the DNA/rAdV immunized sheep (**Figure 7**). This data suggested that both of these two immunization strategies could induce a Th1/Th2 mix type cytokine immune response, while the DNA prime-subunit boost immunization strategy could induce a better response.

## 4 Discussion

Recently, researchers have been working on the development of a safe and effective vaccine for Orf, and there have been some progresses, such as the attenuated vaccine (4) and the DNA vaccine related to the important immunogenic genes of ORFV (15), which have demonstrated effective in eliciting specific antibodies against ORFV. However, attenuated vaccines usually have a risk of virulence reversion (16). DNA vaccines have generally the disadvantage of poor antigenicity (17). So far, no universally approved vaccine has been developed against worldwide strains of ORFV (8, 24). Our previous research has proved that Orf DNA vaccine prime-subunit vaccine boost strategy could enhances the immune response in mice, which gives us an indication of whether other heterologous prime-boost immunization strategies could also lead to stronger immune effect in mice and sheep (22). Exploring the effects of different immunization strategies is essential for accelerating the development of Orf vaccines. In this study, we generated recombinant DNA-based, subunit-based, and rAdV-based ORFV vaccine candidates and performed prime-boost strategies. Then we evaluated the immune responses by heterogeneous (DNA/Protein and DNA/rAdV) immunization in both mice and sheep.

One of the key characteristics of vaccine effectiveness is to induce humoral immune response, which could be positively related to the protective capacity of vaccines (25). Thus, after immunizations of mice and sheep, we evaluated the humoral immune response, our data have found that all vaccines can induce specific IgG for B2L and F1L proteins, and the highest level of specific IgG was found in the DNA/Protein group in both mice and sheep. We have previously shown that DNA prime-subunit boost strategy induced better immune response in mice (22). In the present study, we mainly focused on the comparison of adenovirus-based vaccine (prime-boost strategy) with DNA prime-subunit boost strategy. Previous research found that compared to the homologous vaccination, DNA prime-adenovirus boost vaccination can induce higher specific IgG to porcine reproductive and respiratory syndrome virus GP5 glycoprotein (26) and classical swine fever virus E2 protein in mice (27). However, our data showed that there is no significant difference between DNA/rAdV and DNA vaccine in mice. Our data indicated DNA prime-protein boost strategy could induce stronger specific antibodies than DNA prime-adenovirus boost strategy.

In fact, several studies have also found that DNA vaccine prime-subunit vaccine boost immunization can induce strong IgG antibody than a single-type vaccine. This immune strategy has been reported in multiple viral vaccines, such as HIV (28) MERS-CoV (23), chicken infectious anaemia virus (29), infectious bursal disease virus (30), and Coxsackievirus (31) vaccine research. The reason could be that using DNA vaccine as a primer can not only trigger T cell response, but also stimulate the proliferation and differentiation of antigen-specific memory B cells (28). Compared with DNA vaccine, subunit vaccine can directly stimulate antigen-specific memory B cells to produce antigen-specific antibodies with high titer. Therefore, the subunit vaccine has more advantages in helping to induce good humoral immunity (32). The immunization strategy of DNA prime-protein boost enables the two vaccines to play their respective advantages, which could be better than a single immunization strategy (31). Antibodies were secreted by B cells, which benefit from the production of T helper (Th) cells-stimulating cytokines. Th cells have different subsets, such as Th1 and Th2. The detection of IgG subclass IgG1 and IgG2a levels in serum can be used as an indicator to distinguish the types of Th1 and Th2 responses (33). Our previous study on the ORFV DNA vaccine confirmed that DNA vaccine was more inclined to Th1-type immune response (21). One study has found that subunit vaccine mainly induces Th2-type immune response (34). Similar to these previous studies, through the detection of specific antibody subclasses IgG1 and IgG2a in mice, we found that DNA and DNA/Protein groups could induce a preferential Th1 response, while subunit vaccine could induce a preferential Th2 response.

It is well known that vaccines stimulate the body to produce humoral and cellular immunity, and play an essential role in preventing pathogen infection. T cells are critical in the body’s immune response (35). Animal experimental studies showed that after intraperitoneal inoculation of inactivated ORFV in mice, the expression of Th1 cytokine increased in the middle and early stages, and then Th2 cytokine increased, indicating that T cells play an important role in resisting virus infection (8). Generally, CD4^+^ represents Th cells and CD8^+^ represents Tc cells (killer T cells) (36). CD4^+^T cells, also known as helper T cells, can help CD8^+^T cells to participate in cellular immune killing and to clear virus-infected cells (37). CD4^+^ Th cells can be divided into Th1, Th2 and other cell subsets. Th1 cells mainly secrete IFN-γ, IL-2 and TNF-α; while Th2 cells mainly secrete IL-4 and IL-6, previous studies have found that the Orf DNA vaccine induces a specific T-cell proliferative response (CD4^+^ and CD8^+^) in mice (21). Therefore, to measure the cellular immune response elicited by different immunization regimens, we detected the level of lymphocyte proliferation in mice, and the changes of various cytokines (Th1-type cytokines like IFN-γ, TNF-α and IL-2; Th2-type cytokines like IL-4 and IL-6) in whole splenocytes in mice and sheep by ELISA, the percentage of CD4^+^ and CD8^+^ T cells in the peripheral blood of sheep. In our results, the DNA/rAdV group induced the highest lymphoproliferation responses. The level of Th1 cytokine (IFN-γ, TNF-α, and IL-2) in the DNA/Protein group was significantly higher than that in the DNA group, and the level of Th2 (IL-4 and IL-6) cytokine in the DNA/Protein group was also significantly higher than that in the Protein group. Thus, the DNA prime-protein boost strategy may induce a Th1-biased or a mixed Th1/Th2 response in mice with Th1 and Th2 cytokines production. Similarly, previous studies found that Th1 cytokines play an important role in eliminating ORFV infection, and the increase of CD4^+^cells can also help the body eliminate ORFV (38). In the study for duck enteritis virus, DNA prime-protein boost strategy induced a mixed Th1/Th2 response in mice, and induced a broad humoral and cellular immune response (39). So far, no one has reported the immune response in sheep with the DNA vaccine as the primary immunization vaccine, and the subunit vaccine as the boost vaccine. Our results suggest that DNA prime-protein boost strategy could increase high levels of specific antibodies both in mice and sheep. Importantly, we have also found that DNA prime-protein boost strategy can enhance Th1 and Th2 response in sheep. The percentages of CD4^+^ and CD8^+^ cells in the DNA/Protein are higher than that in DNA/rAdV group. These results further indicate that the combined immunization with DNA and subunit vaccine can induce a T-cell response in mice and sheep. Through our data, we found that the DNA prime-protein boost strategy induced higher levels of humoral and cellular immune responses than DNA prime-adenovirus boost strategy, which may be a meaningful attempt for developing Orf vaccine.

## 5 Conclusion

In conclusion, the heterologous immunization using DNA prime-protein boost strategy induces stronger humoral and cellular immune responses than DNA prime-rAdV boost strategy. Our data provide a new attempt at the exploration of the Orf immunization strategy.

## Data availability statement

The raw data supporting the conclusions of this article will be made available by the authors, without undue reservation. Requests to access these datasets should be directed to Wy987008541@outlook.com.

## Ethics statement

The animal study was reviewed and approved by the Regulations on Requirements for Laboratory Animals and Laboratory Animal Environment and Housing Facilities of China and were approved by the College of Veterinary Medicine of Jilin University.

## Author contributions

JG, KZ and YW participated in designing experiments, YW, SS and KZ performed the experiments, analyzed the data, and wrote the manuscript. JG, KZ, YW, SS, LD, XW, DS, WH and FG revised the manuscript. All authors contributed to the article and approved the submitted version.
